# Leveraging the Tumor Microenvironment as a Target for Cancer Therapeutics: A Review of Emerging Opportunities †

**DOI:** 10.3390/pharmaceutics17080980

**Published:** 2025-07-29

**Authors:** Hakan Guven, Zoltán Székely

**Affiliations:** 1 Robert Wood Johnson Medical School, Rutgers, The State University of New Jersey, 675 Hoes Lane West, Piscataway, NJ 08854, USA; hg373@rwjms.rutgers.edu; 2 Molecular Design and Synthesis Core, Rutgers University Biomolecular Innovations Cores, Office for Research, Rutgers, The State University of New Jersey, 610 Taylor Rd, Piscataway, NJ 08854, USA; 3 Department of Chemistry and Chemical Biology, Rutgers, The State University of New Jersey, 123 Bevier Rd, Piscataway, NJ 08854, USA; 4 Cancer Pharmacology Research Program, Rutgers Cancer Institute of New Jersey, 195 Little Albany St., New Brunswick, NJ 08901, USA; 5 Xiconic Pharmaceuticals, LLC., 31 West 31st Street, New York, NY 10001, USA

**Keywords:** prodrugs, small-molecule–drug conjugate, antibody–drug conjugate, cancer, tumor microenvironment, drug design, systemic toxicity, bystander effect, hypoxia

## Abstract

Cancer has remained one of the leading causes of death worldwide throughout history despite significant advancements in drug development, radiation therapy, and surgery. Traditional chemotherapeutic small molecules are often hindered by narrow therapeutic indices and limited specificity, leading to suboptimal clinical outcomes. On the other hand, more advanced approaches, such as antibody–drug conjugates (ADCs), frequently encounter obstacles, including poor tumor penetration and prohibitive production costs. The tumor-forming and metastatic capacity of cancer further challenges currently available cancer therapies by creating a biochemical milieu known as the tumor microenvironment (TME). Although solid tumor development presents significant obstacles, it also opens new avenues for innovative therapeutic approaches. It is well-documented that as tumors grow beyond 1–2 mm^3^ in size, they undergo profound changes in their microenvironment, including alterations in oxygen levels, pH, enzymatic activity, surface antigen expression, and the cellular composition of the stroma. These changes create unique opportunities that can be exploited to develop novel and innovative therapeutics. Currently, numerous ADCs, small-molecule–drug conjugates (SMDCs), and prodrugs are being developed to target specific aspects of these microenvironmental changes. In this review, we explore five TME parameters in detail, with a focus on their relevance to specific cancer types, phenotypic identifiers, and preferred methods of therapeutic targeting. Additionally, we examine the chemical moieties available to target these changes, providing a framework for design strategies that exploit the dynamics of the tumor microenvironment.

## 1. Introduction

### 1.1. Cancer and the Tumor Microenvironment

Cancer has remained one of the leading causes of death worldwide, affecting many populations across diverse geographies and time periods. Solid tumors, in particular, present a significant therapeutic challenge due to their poor prognosis and complex biological behavior [1]. This complexity arises from the fact that cancer is not merely a cellular disease but a condition profoundly influenced by its surrounding microenvironment. The interplay between neoplastic cells and the surrounding stromal, immune, and vascular components creates a cooperative environment that can autonomously regulate critical physiological parameters, such as metastasis, immune evasion, and drug resistance [2].

The foundation of this complex ecosystem-like view of cancer biology can be traced back to the pioneering work of the surgeon Stephen Paget in the late 1800s. In his observations of breast cancer metastasis, Paget proposed the “seed and soil” hypothesis, suggesting that the distribution of metastatic tumors is not random but rather reflects a pattern of compatibility between the migrating cancer cells and the specific microenvironments of distant organs [3]. This concept has since evolved to encompass a much more detailed understanding of the molecular and cellular constituents of the tumor microenvironment (TME), which supports metastatic growth. Some of these components include endothelial cells, pericytes, fibroblasts, lymphocytes, and macrophages, each of which contributes to the development and support of a hospitable niche for invading tumor cells [4]. Clinically, this phenomenon is so crucial for cancer growth that it is reflected in metastatic patterns, where certain organs and tissues often serve as the “fertile ground” for the growth and proliferation of secondary malignancies [5].

### 1.2. Treating Solid Tumors

**Figure 1:**
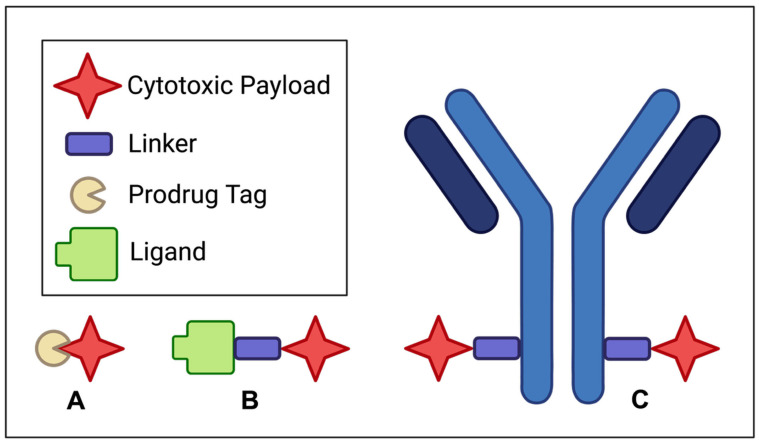
Structural schematics for (**A**) prodrugs, (**B**) small-molecule–drug conjugates (SMDCs), and (**C**) antibody–drug conjugates (ADCs).

The treatment options for solid tumors are diverse, including invasive surgical resection, radiotherapy, targeted therapy, and immunotherapy [6]. To expand the scope and utility of currently available chemotherapeutics, significant efforts are being made in the pursuit of developing prodrugs, small-molecule–drug conjugates (SMDCs), and antibody–drug conjugates (ADCs) for their versatility, specificity, and potential clinical benefits. Figure 1 summarizes the structural motives of the aforementioned therapeutic modalities. 

For instance, prodrugs are designed to be selective with reduced systemic toxicity by chemically masking bioactive agents until they reach their intended targets. This approach establishes a reliable activation mechanism and, therefore, minimizes off-target effects [7]. SMDCs, while structurally distinct from ADCs, achieve the shared goal of targeted drug delivery in addition to offering the advantages of smaller size and, therefore, potentially superior tissue penetration [8]. 

Unlike their larger ADC counterparts, SMDCs can benefit from small-molecule pharmacokinetics to achieve rapid tumor penetration, potentially improving drug accumulation at the target site. In contrast, ADCs represent a highly selective and potent approach to targeting cancer cells, integrating the precision of monoclonal antibodies with extremely cytotoxic molecules. This way, ADCs can deliver potent payloads directly to tumor cells while sparing healthy tissues [9].

However, despite these promising chemical approaches, significant challenges remain regarding our understanding of the biological aspects and pharmaceutical consequences of the TME. One of the most pressing issues is the development of multidrug resistance in solid tumors, which can severely limit the long-term efficacy of these therapies [1]. These clinical observations highlight the need for innovative drug designs to target the TME.

### 1.3. Clinical Perspective of TME

Beyond nurturing the growth of primary cancer cells, TME plays a pivotal role in drug resistance and metastasis [10]. Despite our extensive arsenal of chemotherapeutic agents, cancer treatment remains less effective than many conventional therapies for other diseases, particularly in the context of solid tumors, where the approach, in many cases, is palliative rather than curative [11].

Solid tumors present certain unique physical barriers to drug delivery, including dense, fibrous capsules that compress blood vessels and lymphatic conduits, reducing drug penetration and nutrient exchange [12]. This physical constraint disrupts the delicate balance of Starling forces that typically drive fluid and solute exchange at the capillary level, creating an antagonistic environment for effective drug delivery. Furthermore, the TME harbors a wide range of biological features that contribute to therapeutic resistance and disease progression. These include a variety of mechanisms, from anti-apoptotic signaling pathways to metabolic alterations [13]. In fact, it has been demonstrated in animal models that the behavior and drug resistance of cancer cells are dictated by the location of the tumor and its microenvironment [14]. Some resistance mechanisms and cancer phenotypes can only be observed in vivo, which further highlights the importance of TME in cancer biology and treatment response [15].

These findings emphasize the need for a multifaceted approach toward developing solid tumor therapeutics that consider the intrinsic properties of cancer cells as well as the microenvironments in which they reside. This perspective will be critical for the design of next-generation therapies capable of overcoming the formidable barriers posed by the TME.

### 1.4. Objectives

The foundational approach to cancer treatment has traditionally relied on identifying the key biochemical distinctions between healthy and neoplastic cells and using these insights to develop innovative therapies [16]. However, this strategy alone has proven insufficient, as more than 90% of cancer drugs fail in clinical trials, often due to the limited ability to accurately model solid tumors in a laboratory setting [17]. This challenge highlights the critical need for a paradigm shift in cancer drug discovery to target the supportive TME. This review aims to provide a discussion of the various TME attributes that could serve as viable targets, offering insights for scientists across a multitude of diverse disciplines to elevate the importance of systems biology. Ultimately, this paper aims to serve as a thought-provoking guide to designing ADCs and seeks to foster a collaborative, multidisciplinary approach to ADC design. 

## 2. TME Properties

### 2.1. Genesis and Constituents

**Figure 2:**
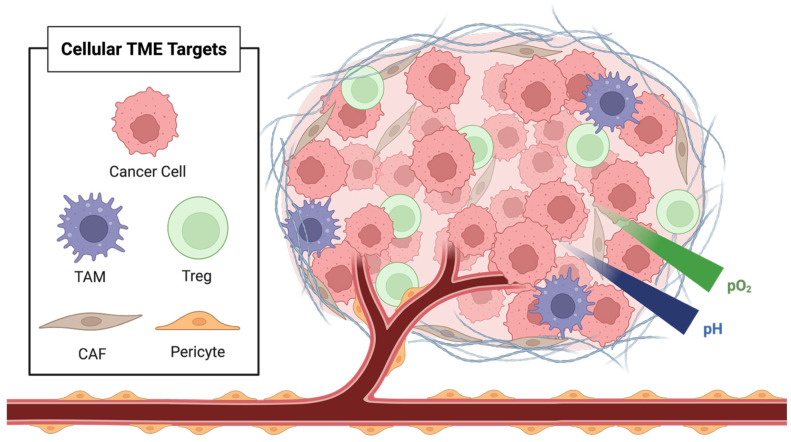
Cellular components and biochemical characteristics of the tumor microenvironment (TME), including cancer cells, tumor-associated macrophages (TAM), regulatory T cells (Treg), cancer-associated fibroblasts (CAF), and pericytes.

TME originates with the emergence of a mutant cell population. While neoplastic transformation arises from intrinsic cellular alterations, tumor cells depend on signals from a supportive microenvironment to sustain proliferation and malignant progression. If these transformed cells arise in a permissive environment, they initiate reciprocal signaling with the surrounding stromal tissue, thereby setting the stage for early TME development [18]. This interaction is mediated by small molecules, secreted growth factors, and chemokines orchestrated through paracrine and autocrine signaling between tumor cells and the resident tumor-associated immune cells [19]. The cellular residents of the TME can be broadly classified into three functional groups: vascular, immune, and structural; each contributes uniquely to tumor progression by altering the biochemical and immunological milieu around cancer cells. Figure 2 depicts the complex cellular infrastructure of the TME.

One of the earliest and most critical signals in TME development is the induction of angiogenesis and lymphangiogenesis. This process is mediated by signaling molecules such as fibroblast growth factor (FGF), vascular endothelial growth factor (VEGF), and platelet-derived growth factor (PDGF), all of which are present in the early TME and are essential for establishing blood and lymphatic conduits that support tumor nutrition and metastatic dissemination [20,21]. As the vascular infrastructure expands, the TME begins to diversify, incorporating a wide range of both cellular and non-cellular components that enable tumor survival and proliferation. These include immune cells such as regulatory T cells (Tregs) and tumor-associated macrophages (TAMs), as well as endothelial cells, pericytes, and cancer-associated fibroblasts (CAFs) [22,23].

Among the various immune modulators in the TME, Tregs stand out as key architects of immune tolerance. Although Tregs may have varied effects across cancer types, in the context of solid tumors, they are critical for dampening anti-tumor immunity and impairing the adaptive immune response to tumor-associated antigens [24,25]. These cells express potent immunosuppressive mediators, including IL-10, TGF-β, and CTLA-4, which collectively suppress the maturation and activity of cytotoxic T cells, thereby facilitating immune evasion by tumor cells [26,27]. TAMs also play a vital role in shaping the TME, particularly in promoting angiogenesis. As tumors enlarge and hypoxia intensifies due to poor vascularization, TAMs respond by upregulating pro-angiogenic factors that effectively control the angiogenic switch, allowing tumors to survive and grow under stress [28].

The increasing tumor mass and the formation of a fibrotic capsule are major drivers of tumor hypoxia that restrict adequate vascular perfusion [29]. Large contributors to this fibrotic environment are the CAFs, which play a central role in both the physical architecture and signaling throughout the TME. CAFs are responsible for secreting mitogenic and pro-angiogenic factors, as well as for synthesizing and cross-linking dense extracellular matrix (ECM) proteins, such as collagen and elastin [30]. Beyond remodeling, CAFs also secrete a broad range of growth factors, including hepatocyte growth factor (HGF), fibroblast growth factor (FGF), insulin-like growth factor 1 (IGF-1), and TGF-β, which drive tumor cell proliferation and induce epithelial-to-mesenchymal transition [31,32].

Overall, the TME represents an interwoven system of tumor and non-transformed host cells engaged in constant communication through a dynamic network of chemokines, cytokines, and growth factors. Understanding this networking is essential for designing therapies that interrupt the cumulative tumorigenic effects of the TME. 

### 2.2. Pharmacological Consequences

The TME plays a central role in driving immune evasion, promoting drug resistance, and facilitating metastasis, which are the three key contributors to solid tumor aggressiveness and ultimately, therapeutic failure [20,33]. The various anti-immune cytokines and the activation of Tregs contribute to immune evasion by preventing tumor cells from being recognized and destroyed by the immune system. This immune suppression affects not only endogenous immune mechanisms (innate and adaptive) but also reduces the efficacy of exogenous immunotherapies used in the clinic [34]. The TME also induces the upregulation of various immune checkpoint ligands on cancer cells, leading to decreased activity of immune cells such as T cells and NK cells. These checkpoint receptor–ligand interactions, which normally serve to limit autoimmune responses, become dysregulated within the TME and contribute to a suppressed immune response against tumor cells [35,36]. Overall, the TME profoundly modulates the way the immune system recognizes and responds to cancer. The problem of immune evasion is further complicated by the physical architecture of most solid tumors [37]. The fibrous capsule and dense ECM produced by CAFs create a proteinaceous barrier that hinders drug penetration and immune cell infiltration. Within this encapsulated zone, dysregulated pH and decreasing oxygen gradients further compromise therapeutic efficacy. As a result, resistance arises not only to immunotherapies but also to drug conjugates and small-molecule chemotherapeutics [38,39].

When we examine the preferred destinations for tumor metastasis, clear patterns emerge. This organ-specific phenomenon is attributed to the presence of tissue environments that are primed with growth factors, permissive biophysical conditions, and supportive stromal cell populations [40]. For instance, prostate and breast cancers frequently metastasize to bone, where they are drawn by chemoattractants like osteopontin and CXCL12, as well as the acidic pH, high calcium concentration, and availability of growth-promoting factors [41]. This idea is further supported as the disruption of the CXCL12/CXCR4 axis has been shown to impair breast cancer metastasis in preclinical models [42]. Similarly, the lungs provide an ideal site for metastatic outgrowth due to their vast vascular surface area [43]. Additionally, in the liver, colorectal cancer cells exploit selectin-mediated adhesion by using sLeX ligands to bind to E-selectin-expressing endothelium, which is commonly found in hepatic tissues [44,45,46]. Finally, in the brain, metastatic cancer cells benefit from the potential to create protective gap junctions with astrocytes and from the immune-deficient environment conferred by the blood–brain barrier, which allows them to escape immunologic clearance [47,48].

**Figure 3:**
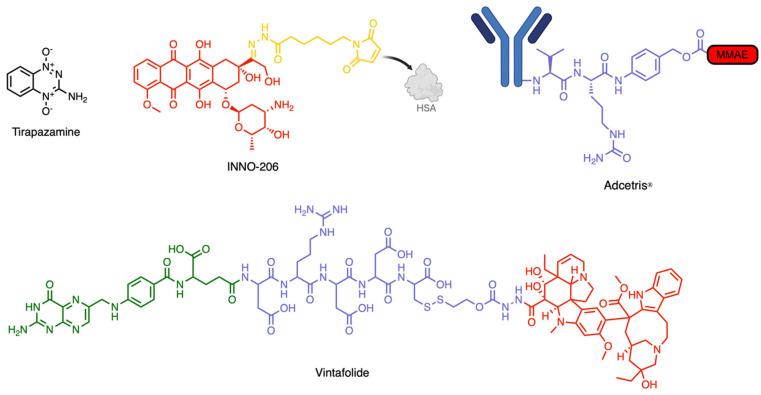
Examples of prodrugs, SMDCs, and ADCs targeting the TME. Tripazamine is a hypoxia-activated prodrug. INNO-206 is a pH-activated prodrug that binds human serum albumin (HSA) in circulation. Adcetris is an ADC that is activated by cathepsin B. Vintafolide is an SMDC targeting the folate receptor alpha (FRα).

Given the biochemical patterns and clinical phenomena discussed above, it is essential to emphasize that the TME is just as critical a therapeutic target as the tumor cells themselves. In fact, dismantling the TME alone may unlock broadly applicable treatment strategies capable of overcoming the challenges posed by solid tumors, enabling the repurposing of clinically failed therapeutics. Figure 3 illustrates various subtypes in molecular design that utilize the TME for enhanced therapeutic effect.

## 3. Tissue Targeting

### 3.1. Hypoxia

Hypoxia is defined as inadequate tissue oxygenation and is a major hallmark of solid tumor growth. This phenomenon arises due to abnormally developed tumor vasculature, heterogeneous spatial disorganization, and increased dense connective tissue remodeling, all of which impair tissue permeability and oxygen diffusion [19,49]. As the tumor mass expands beyond the diffusion limit of oxygen (estimated at approximately 100–200 µm from the nearest capillary), central regions of the tumor become profoundly hypoxic [50]. This type of spatial restriction defines the biochemical heterogeneity between the tumor periphery and its core, serving as the basis of innovative drug design. 

These hypoxic zones are not only a major hallmark of the cell biology of aggressive tumors but are also associated with a lower proliferative index, which protects cancer cells by decreasing their susceptibility to conventional therapies such as chemotherapy and radiotherapy that target rapidly dividing cells [51]. As a result, therapeutic failure in hypoxic tumors is common, which highlights the importance of developing novel strategies that bypass cell cycle dependence and selectively act under such conditions [52,53]. 

As a clinical manifestation, hypoxia is especially prominent in carcinomas of the cervix, head and neck, and lungs, with studies showing that approximately 50% of cervical tumors and their metastatic nodules exhibit severe oxygen deprivation [38,54]. However, hypoxia is not exclusive to these subtypes and can occur in various solid tumors. Recent advances in tumor hypoxia imaging (such as Eppendorf oxygen electrodes and PET using nitroimidazole tracers) can enable clinicians to assess hypoxia at the individual patient level, thereby facilitating more precise treatment decisions [55]. 

#### Hypoxia-Activated Prodrugs 

Therapeutic application to exploit hypoxia in tumors currently focuses on hypoxia-activated prodrugs (HAPs): small molecules structurally engineered to remain safe and inert under normoxic conditions but can become selectively cytotoxic in hypoxic tissues. These molecules typically incorporate bioreducible chemical moieties, including quinones, nitroaromatics, and N-oxides [56]. Once within hypoxic tumor microenvironments, these functional groups undergo enzymatic activation, typically *via* reductases such as cytochrome P450, ferredoxin, and quinone oxidoreductases, which catalyze electron transfer into the prodrugs, generating active chemical entities that are cytotoxic [57,58]. These reduced metabolites can form DNA-damaging species, such as hydroxyl radicals or alkylating intermediates, resulting in tumor cell death [59,60]. 

Although no HAPs have yet received FDA approval, several candidates have shown strong preclinical performance and have entered clinical trials. One of the earliest and most studied HAPs is tirapazamine, which set the precedent for selective hypoxia targeting. Tirapazamine undergoes one-electron reduction under hypoxic conditions to produce a toxic radical intermediate that induces DNA double-strand breaks [61,62]. This activity is highly selective; under hypoxic conditions, only 1–2% of the dose under normoxic conditions is needed to achieve equivalent cytotoxicity [63,64]. 

The efficacy of tirapazamine as a proof-of-concept in preclinical models has spurred further research into next-generation HAPs such as PR-104, EO9, and TH-302, utilizing novel prodrug structures that include a reductase-dependent activation pathway [56,59,65]. These agents have been investigated across a variety of tumor models, including sarcoma, head and neck cancers, and colorectal tumors, given their hypoxic nature. While challenges such as intra-tumor heterogeneity, drug penetration, and metabolic variations across patients persist, the HAP model remains one of the most biochemically relevant strategies for utilizing the TME in drug design.

### 3.2. pH Imbalance 

Compared to the physiological pH of extracellular tissues, which is 7.4, solid tumors generally maintain a significantly lower pH, ranging from about 6.5 to 7.0. This acidotic change is mainly driven by the Warburg effect, a major metabolic adaptation in tumor cells that favors aerobic glycolysis, converting glucose to lactate even under normoxic conditions [66]. As a result of this metabolic switch, large amounts of lactic acid and protons accumulate in the TME, leading to chronic extracellular acidosis. This acidic niche supports several cancer-enabling pathways, including anabolic metabolism, resistance to apoptosis, and protection from oxidative stress [67].

It is essential to note that tumor acidosis is not merely a passive consequence of uncontrolled growth but rather an active regulator and promoter of overall malignancy [68]. The low pH leads to tissue invasion by increasing extracellular matrix (ECM) degradation and promoting phenotypic changes that support metastasis. Furthermore, it has been described in the literature that this pH imbalance creates an immunosuppressive microenvironment for macrophages, T cells, and NK cells [69]. 

The acidic TME has been observed across multiple different cancer subtypes such as glioblastoma, melanoma, colorectal, pancreatic, prostate, and triple-negative breast cancer [69,70,71]. However, it is important to mention that pH imbalance is not exclusive to the previously mentioned cancer types but is a potential attribute in any solid tumor. Therefore, advancements in imaging technologies that can allow clinicians to determine the pH balance of tumor cells could prove helpful in a clinical setting where drug administration could be guided by the extent of TME presence. In fact, pH imaging can be performed using PET tracers, magnetic resonance spectroscopy (^31^P, ^13^C, ^19^F), or fluorescent probes, such as BOPIDY and cyanine dyes, each offering insight into TME acidity [72,73]. 

#### pH-Activated Molecular Systems

The potential of pH gradients as a biochemical target has been explored directly in cancer pharmacology and indirectly in organic synthesis through the design of acid-sensitive moieties, prodrugs, and cleavable linkers. One of the most direct methods for exploiting tumor acidosis is ion trapping, a biophysical phenomenon where weakly acidic and lipophilic drugs accumulate in the acidic extracellular space of tumors [74]. This is largely dependent on the drug molecules’ pKa matching the pH gradient of the TME. For instance, chlorambucil (pKa 5.8) has demonstrated increased uptake in acidic conditions, with various studies showing a 3.6- to 4.5-fold increase in drug concentration under tumor-simulating conditions, thereby substantially enhancing its cytotoxic potential [75]. 

More sophisticated approaches for targeting the pH gradient involve acid-activated prodrugs, which utilize cleavable linkers to release cytotoxic agents selectively under acidic environments [76,77]. For example, doxorubicin (DOX) analogs have been conjugated to hydrazone-based linkers that dissociate in mildly acidic locations, enabling controlled and tumor-specific drug activation [78]. INNO-206, a pH-activated DOX conjugate, bound to HSA after IV administration, has shown increased therapeutic efficacy by passive targeting [79]. In fact, these chemical motifs have shown such promise that similar acid-sensitive designs have been used in hydrogel systems for broader biomedical applications [80].

A third emerging strategy focuses on pH-sensitive liposomes and nanoparticles. These carriers remain stable in the bloodstream but destabilize under acidic conditions, potentially releasing their payload in a more effective manner within the TME. Liposomes incorporating pH-sensitive lipids such as dioleoylphosphatidylethanolamine (DOPE) exhibit enhanced membrane fusion and content release at low pH, showing promising preclinical results for targeted drug delivery [81,82]. Although these systems have not yet been widely adopted in oncology, they offer strong potential for clinical translation due to their specificity and reduced systemic toxicity. 

## 4. Cellular Targeting

### 4.1. Proteolytic Enzymes 

The TME has a distinct enzyme composition compared to adjacent healthy tissues, which are primarily shaped by reciprocal signaling between tumor cells and the vicinal stromal niche. This enzyme composition varies in both identity and expression level. Among these enzymes, proteases are of particular interest for their catalytic activity of cleaving covalent bonds to activate molecular entities. In addition to their utility in drug activation, several enzymes within the TME, such as cathepsin B, heparanase, and lysyl oxidase (LOX), have also been explored as direct therapeutic targets. Inhibitors like CA-074 (cathepsin B), PI-88 (heparanase), and various integrin and LOX antagonists have shown potential to suppress tumor growth and proliferation in preclinical models [83,84,85,86,87,88,89,90].

Matrix metalloproteinases (MMPs), particularly MMP-2 and MMP-9, have been studied extensively for their role in cancer biology. Elevated expressions of these extracellular proteases have been detected in a wide range of solid tumors, including breast, pancreatic, lung, bladder, and colorectal cancers [91]. MMPs’ proteolytic activity facilitates proliferation, angiogenesis, and metastasis, correlating with higher malignancy risk and serving as a biomarker for advanced tumor grading [92,93]. Similarly, cathepsin B, an intracellular lysosomal cysteine protease, is markedly overexpressed in breast, thyroid, and colorectal cancers [94,95,96]. Given the critical role these proteases play in tumor proliferation and progression, their detection has become essential for both diagnostic purposes and guiding treatment strategies [97]. Beyond the more traditional techniques such as immunohistochemistry (IHC) and ELISA, recent imaging advancements have allowed the non-invasive visualization of protease activity in vivo. Tools such as protease-activated fluorescent probes and MRI substrates have demonstrated utility in dynamically mapping enzymatic densities [72,91,98]. These modalities are particularly valuable for tumors with spatially heterogeneous enzyme expression, such as glioblastoma, invasive breast cancer, and metastatic thyroid cancer, for clinicians to determine a treatment course [99]. 

#### Proteolytically Activated Therapeutics

As the overall enzymatic activity of tumor-associated proteases increases, a unique opportunity arises for drug development teams to design chemical agents that are selectively activated within the TME. This principle has already been applied in the design of prodrugs and ADCs, where enzyme-cleavable linkers ensure that cytotoxic payloads are released specifically at the tumor site.

MMPs commonly target the ECM collagen and exhibit a relatively broad substrate specificity. A frequently observed cleavage motif includes the sequence Pro-X-X-Hy-(Ser/Thr) (Hy is a hydrophobic residue), which mimics the natural collagen cleavage as well as other ECM peptides [100]. This lack of substrate specificity has been exploited in drug design by conjugating cytotoxic agents with cleavable peptide linkers that are activated by MMPs. For instance, HT1080, a DOX analog, conjugated with the MMP-cleavable motif Ac-γGlu-Pro-Cit-Gly-Hof-Tyr-Leu, demonstrated an improved therapeutic index while maintaining its efficacy in preclinical xenograft models [101].

Similarly, cathepsins (particularly cathepsin B) represent a key enzymatic target due to their high expression and activity in the TME [102]. Unlike MMPs, which function extracellularly, cathepsin B is a lysosomal protease, enabling the selective activation of prodrugs and ADCs within tumor cells. Cathepsin B is quite promiscuous in its binding site and cleaves a variety of substrates, including Phe-Lys, Ala-Leu-Ala-Leu, and Gly-Phe-Leu-Gly, all of which have been used to cage DOX and other cytotoxic agents in various prodrug designs [103,104]. However, the most widely adopted motif for cathepsin B cleavage in ADCs is the dipeptide Val-Cit, prized for its balance of plasma stability and rapid lysosomal cleavage [105,106]. This linker has been incorporated into several FDA-approved ADCs, triggering the release of cytotoxic payloads upon endocytosis and lysosomal activation. 

### 4.2. Surface Antigens 

Perhaps one of the most clinically actionable features of the TME is the overexpression of surface antigens that are accessible and available to therapeutic agents. From a drug design perspective, the most valuable targets are typically those that are highly overexpressed and tumor-specific. These can be broadly classified into two categories: signaling receptors and nutrient transporters. Prominent examples include HER2, TROP2, folate receptor alpha (FRα), glucose transporter 1 (GLUT1), and various angiogenic receptors, all of which are not only associated with tumor growth and survival but also correlate with poor prognosis and increased metastatic potential [107,108,109,110,111].

For instance, HER2 overexpression is observed in approximately 30% of breast and ovarian cancers, and it is associated with increased aggressiveness and treatment resistance [107,112]. Similarly, FRα is upregulated in triple-negative breast cancers and certain ovarian cancers, providing a metabolic growth advantage through enhanced folate uptake [109]. Lastly, GLUT1, a facilitative glucose transporter, is also frequently upregulated in highly glycolytic tumors such as head and neck as well as breast cancers, where it serves both as a diagnostic biomarker and a prognostic indicator [111].

Furthermore, it is worth noting that these surface antigens are often not exclusive to a specific type of cancer. Their consistent and measurable expression could allow clinicians to identify suitable targets on a patient-by-patient basis using established techniques such as IHC, fluorescence in situ hybridization, immunofluorescence, and flow cytometry [112]. All of these developments enable the exploration of precision-targeted therapies that can be tailored according to surface marker expression.

#### Cell Surface Targeting

The therapeutic exploitation of overexpressed surface antigens has driven the development of multiple drug delivery platforms, including SMDCs, ADCs, and monoclonal antibodies (mAbs). These systems rely on the signaling capability of targeting ligands or antibodies to deliver cytotoxic payloads directly to tumor cells, thereby minimizing off-target effects and widening the therapeutic index [113]. There are many other approaches that target unique ligands on cell surfaces, such as immune checkpoint inhibitors, which block receptors on immune cells to prevent immunosuppression, and CAR-T and CAR-NK cell therapies, which program a patient’s own immune cells to recognize specific surface antigens [36,114]. However, in this section, we focus on surface targeting strategies that exploit antigens as homing devices for therapeutic delivery.

A landmark example of this mode of targeting is trastuzumab emtansine (T-DM1), an ADC that consists of a HER2-targeting trastuzumab with a microtubule inhibitor, enabling the precise delivery of cytotoxic therapy to HER2-overexpressing tumor cells while avoiding physiologically healthy tissue that does not express HER2 [115,116].

SMDCs achieve similar tumor surface antigen specificity without the use of antibodies. For instance, vintafolide, a conjugate of folic acid and desacetyl vinblastine, targets FRα-overexpressing tumors through ligand-directed delivery. The previously inert SMDC is uptaken by cells and the disulfide ester linker is digested, yielding the active form and effectively causing apoptosis [8,117]. Despite setbacks in its Phase III study, this compound has provided the drug design community with a strong proof-of-concept for SMDCs [118,119]. Together, these targeting strategies illustrate the therapeutic promise of exploiting surface antigen overexpression. 

### 4.3. Stromal Niche 

In addition to malignant tumor cells, the TME contains a diverse, supportive stromal cell network that plays a crucial role in tumor proliferation, immune suppression, and therapeutic resistance. Because the individual components and consequences of the stromal niche were discussed in detail earlier, this section will focus on its therapeutic potential. The stromal niche established through paracrine and autocrine signaling loops is indispensable for the survival and progression of many solid tumors and, therefore, warrants in-depth study [120,121,122]. 

#### Peripheral Disruption

Targeting stromal components of the TME offers a promising avenue to improve treatment efficacy, particularly in cases where conventional therapies are undermined by drug resistance, immune evasion, or physical barriers. Much like targeting cancer cells, stromal targeting begins with identifying cell-specific enzymatic or surface receptors and designing therapeutics to selectively engage them.

Among the most extensively studied stromal targets are CAFs. Two key surface markers, such as fibroblast activation protein and GPR77, have been identified as candidate targets on CAFs [123,124,125]. These markers may potentially be exploited by SMDCs, ADCs, or mAbs to selectively eliminate tumor-supportive fibroblasts. However, it is essential to acknowledge the challenges of targeting CAFs, as they present unique obstacles due to their heterogeneous distribution within the TME and the absence of specific surface markers for identification [122,126]. As a result, their value may be best realized in combination strategies rather than as stand-alone approaches.

A particularly promising idea of stromal targeting lies in the selective destruction of immune cells within the TME, especially TAMs and Tregs. These cell types have been linked to poor clinical outcomes due to their roles in immunosuppression, angiogenesis, and cytokine-driven tumor progression [120,127]. The clinical success of ipilimumab, an anti-CTLA-4 mAb, underscores the viability of this strategy. By depleting CTLA-4-expressing Tregs in the TME via antibody-dependent cellular cytotoxicity, ipilimumab enhances anti-tumor immune responses and was the first immune checkpoint inhibitor to gain FDA approval in 2011 [128,129,130]. Other mAbs, such as anti-CCR4 (e.g., mogamulizumab), aim to block Treg trafficking into the tumor, although clinical results have been mixed [131]. We hypothesize that effectively targeting the various stromal components of the TME will ultimately enhance the therapeutic efficacy of primary agents such as ADCs, SMDCs, and prodrugs. Reducing the presence of CAFs, TAMs, and Tregs could improve drug penetration and decrease resistance to therapeutics. 

## 5. Current Limitations of ADC Designs

### 5.1. Obstacles to ADC Success

ADCs represent the paramount achievement in cancer drug development. Their specificity is determined by the mAb, their stability and release/activation mechanism are maintained by the linker, and their potency is provided by the cytotoxic payload, collectively offering a blueprint for precision treatment with reduced off-target toxicity. However, clinical outcomes have shown that ADCs still suffer from many of the limitations of conventional therapeutics, presenting several drawbacks in their application [132].

#### 5.1.1. Antigen Availability

A key challenge in ADC development is the limited availability of tumor-specific antigens on cell surfaces. Ideal surface targets should be abundantly expressed (i.e., >100,000 copies) on cancer cells while being absent or very minimally expressed in normal tissues to ensure specificity without toxicity [133]. This challenge is reflected in the relatively narrow pool of antigens that are shared among FDA-approved ADCs, most of which target just a handful of antigens [134]. Another key consideration for targeting solid tumors with ADCs is the dynamic nature of the TME, which evolves over time and in response to various treatments. Following radiation or chemotherapy, the TME adapts by increasing proinflammatory cytokine production, promoting an activated phenotype in CAFs and TAMs, and inducing antigen escape [10]. Exacerbating this issue is the heterogeneity in antigen expression, which is present not only across patient populations but also within the same tumor. This variability represents both a biological obstacle and a mechanism of acquired resistance, where tumors may downregulate, shed, or mutate surface antigens, allowing escape from ADC recognition under therapeutic pressure [135]. Such dynamic alteration of the antigen fingerprint/map undermines therapeutic efficacy and narrows the clinical utility of ADCs [136].

#### 5.1.2. Tumor Penetration

Beyond the challenge of antigen selection, the physical delivery of ADCs into solid tumors presents an unforgiving barrier. Unlike hematologic malignancies, where malignant cells are directly accessible through circulation, solid tumors are protected within their microenvironment; not surprisingly, this has led to an earlier approval of leukemia- and lymphoma-targeting ADCs. Attributes such as abnormal vasculature, dense stromal architecture, and high interstitial pressure collectively hinder ADC penetration and prevent uniform drug distribution [137]. This challenge is further complicated by the so-called “binding site barrier,” where ADCs are sequestered by the first layer of antigen-expressing tumor cells near vasculature, limiting further diffusion into deeper tumor regions [138]. Over time, this leads to a concentration gradient across the tumor, with the periphery receiving therapeutic doses and the core remaining largely untreated. 

#### 5.1.3. Systemic Toxicity

Perhaps the most frequently encountered problem in ADC design is the issue of premature linker activation, which results in payload release in circulation or non-target tissues [139]. While ADCs are engineered for greater specificity than conventional chemotherapies, systemic toxicity remains a key limitation. This is due to the current challenges in designing linkers that are selectively activated only at the intended tumor site [140]. In contrast to the bystander effect (which may offer therapeutic advantages despite its uncontrollability), systemic toxicity is better understood as a pharmacokinetic phenomenon based on random drug activation.

### 5.2. Bystander Effect

The bystander effect refers broadly to the unintended death of nearby antigen-negative cells that are not themselves neoplastic [141]. This phenomenon is a byproduct of the complex biology of cancer cells and holds the potential for therapeutic leverage in ADC design. Unlike systemic toxicity (which reflects widespread exposure), the bystander effect manifests as local toxicity, depending on the ADC’s specific chemical and biological properties.

The major contributor to the bystander effect is the persistent activity of cytotoxic payloads following ADC degradation. As mentioned earlier, these payloads are intentionally chosen to be thousands to tens of thousands of times more potent than traditional chemotherapeutics since only a small fraction of the ADC molecular weight is the payload [142]. However, this extreme potency can pose a liability if the toxin is not rapidly degraded after inducing initial cell death. It may diffuse into adjacent normal cells and tissues, triggering unintended damage. Clinically, such off-target activity has been implicated in many ADC-related adverse events, which are often attributed to the toxicity of the payload on healthy tissue [143,144].

**Figure 4:**
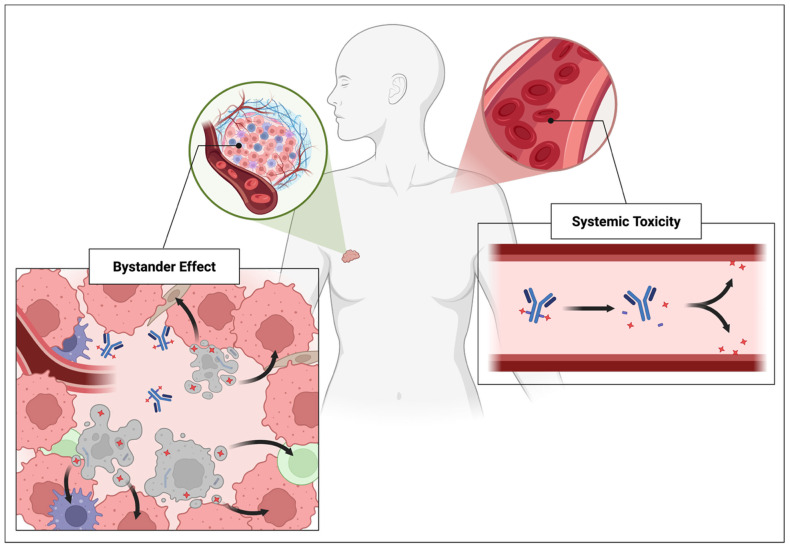
Different payload release mechanisms resulting in the bystander effect or systemic toxicity.

A second contributor to the bystander effect, though less frequently discussed, is a consequence of non-processed ADCs. These refer to ADCs that are not internalized or metabolized as intended, often due to insufficient antigen density or inefficient internalization kinetics [145]. The precise biochemical mechanisms behind this phenomenon remain unclear, but it is hypothesized that non-processed ADCs may persist in circulation and release their cytotoxic payload in unintended locations. As a result, they can contribute to both systemic toxicity and the bystander effect [145]. Figure 4 emphasizes the fundamentally distinct fates of ADCs that lead to bystander effect or systemic toxicity. 

It is clear from both preclinical and clinical findings that the bystander effect is not entirely detrimental and may, in fact, be advantageous if properly harnessed. For instance, the bystander effects observed with trastuzumab deruxtecan (T-DXd) and trastuzumab duocarmazine (SYD985), as well as ADCs incorporating caspase-3-cleavable linkers, have demonstrated enhanced efficacy with intratumoral antigen heterogeneity [146,147]. Thus, rather than being seen solely as a drawback, the bystander effect represents a potential advantage.

## 6. Conclusions

For ADCs to reach their full therapeutic potential, several cellular and environmental characteristics of the TME must be considered during the design phase. In addition, the development of ADCs and other therapeutic platforms must proceed in an iterative manner, with both sides guiding the exploration of the other. SMDC development, in particular, must closely follow the conceptual trajectory laid out by ADCs, as both face similar obstacles, such as the limited number of ideal surface antigens and lack of robust linker designs [148,149]. As SMDCs begin to adopt more cytotoxic payloads more commonly, the lessons from ADCs regarding robust and safe linker strategies must be applied. 

However, linker stability is a double-edged sword, and while it is essential for minimizing off-target effects, it may limit the benefits of the bystander effect. Next-generation ADC designs must strike a balance by incorporating TME-specific activation mechanisms and maintaining high payload potency, especially in tumors with heterogeneous antigen expression. TME-focused strategies can also benefit from combination therapies. Pretreatments with enzymes for stromal connective tissue ablation, mAbs to bypass the binding site barrier or scavenge previously shed antigens, and combined or bispecific ADCs that target the stromal niche may be clinically efficacious [17,150,151,152]. Table 1 summarizes the emerging possibilities in designing novel therapeutics in the context of our growing knowledge of the TME.

As the realm of cancer therapies expands, a shift in target selection from primary tumor cells to the complicit residents of the TME may be transformative. There are many potential avenues to explore, given the complexity of the biological space, but there is also an abundance of chemical moieties to fit that space. Viewing the TME not as an obstacle but as an opportunity, future generations of drug conjugates may unlock a new paradigm in oncology.

## Figures and Tables

**Table 1:** Projected trends of TME targets to currently existing therapeutic modalities.

Therapeutic Modality	Tumor Microenvironmental Factors				
	Tissue Level Targeting		Cellular Level Targeting		
	Hypoxia	pH Imbalance	Enzymes	Antigens	Stromal Niche
ADC	Hypoxia-sensitive linkers	Acid labile pro-ADCs Acid-sensitive linkers	Enzyme cleavable linkers	Combinational or bispecific antigen targeting	Unique homing mAbs
SMDC	Hypoxia-sensitive linkers Hypoxia-activated payloads	Acid-sensitive linkers	Enzyme cleavable linkers	Combinational or bispecific antigen targeting	Unique homing ligands
Prodrug	HAPs	pH-activated prodrugs pH-sensitive liposomes	Enzyme-activated prodrugs	N/A	Novel activation enzymes

## Data Availability

No new data were created or analyzed in this study. Data sharing is not applicable to this article.
